# Female genital mutilation and women’s healthcare experiences with general practitioners in the Netherlands: A qualitative study

**DOI:** 10.1371/journal.pone.0235867

**Published:** 2020-07-07

**Authors:** Ramin Kawous, Emily Allwood, Evelien Norbart, Maria E. T. C. van den Muijsenbergh

**Affiliations:** 1 Department of Public Health, Erasmus University Medical Center, Rotterdam, The Netherlands; 2 Pharos, Dutch Centre of Expertise on Health Disparities, Utrecht, The Netherlands; 3 Department of Primary and Community Care, Radboud University Medical Centre Nijmegen, Nijmegen, The Netherlands; Università degli Studi di Perugia, ITALY

## Abstract

**Objectives:**

While the general practitioner (GP) in the Netherlands is the first point of entry to and gatekeeper of the healthcare system, no study exists to explore the experiences of women with female genital mutilation or cutting (FGM/C) in general practice. Therefore, the aim of this study is to look into the experiences of women with FGM/C in Dutch general practice.

**Methods:**

Semistructured interviews were held with 16 women with FGM/C. Sampling was purposeful. The interview guide and thematic analysis were based on the Illness Perception Model and Kleinman’s Explanatory model. Interviews were held in English or Dutch. All data were anonymized, and recordings were transcribed verbatim. Transcripts were coded and thematically analyzed.

**Results:**

The women considered FGM/C to be connected to a range of health problems, for which not all of them sought medical care. They had difficulty discussing such a sensitive topic with their GP, did not know their problems could be relieved or perceived GPs to have insufficient knowledge of FGM/C. Lack of time during consultations and overall dissatisfaction with Dutch GP care hampered trust. They strongly preferred the GP to be proactive and ask about FGM/C.

**Conclusion:**

There is room for improvement as most women would like their GP to discuss their health problems related to FGM/C. GPs should take a proactive attitude and ask about FGM/C. In addition, to develop the trusted relationship needed to discuss sensitive topics and provide culturally sensitive person-centered care, sufficient time during consultations is needed.

## Introduction

**Fig 1:**
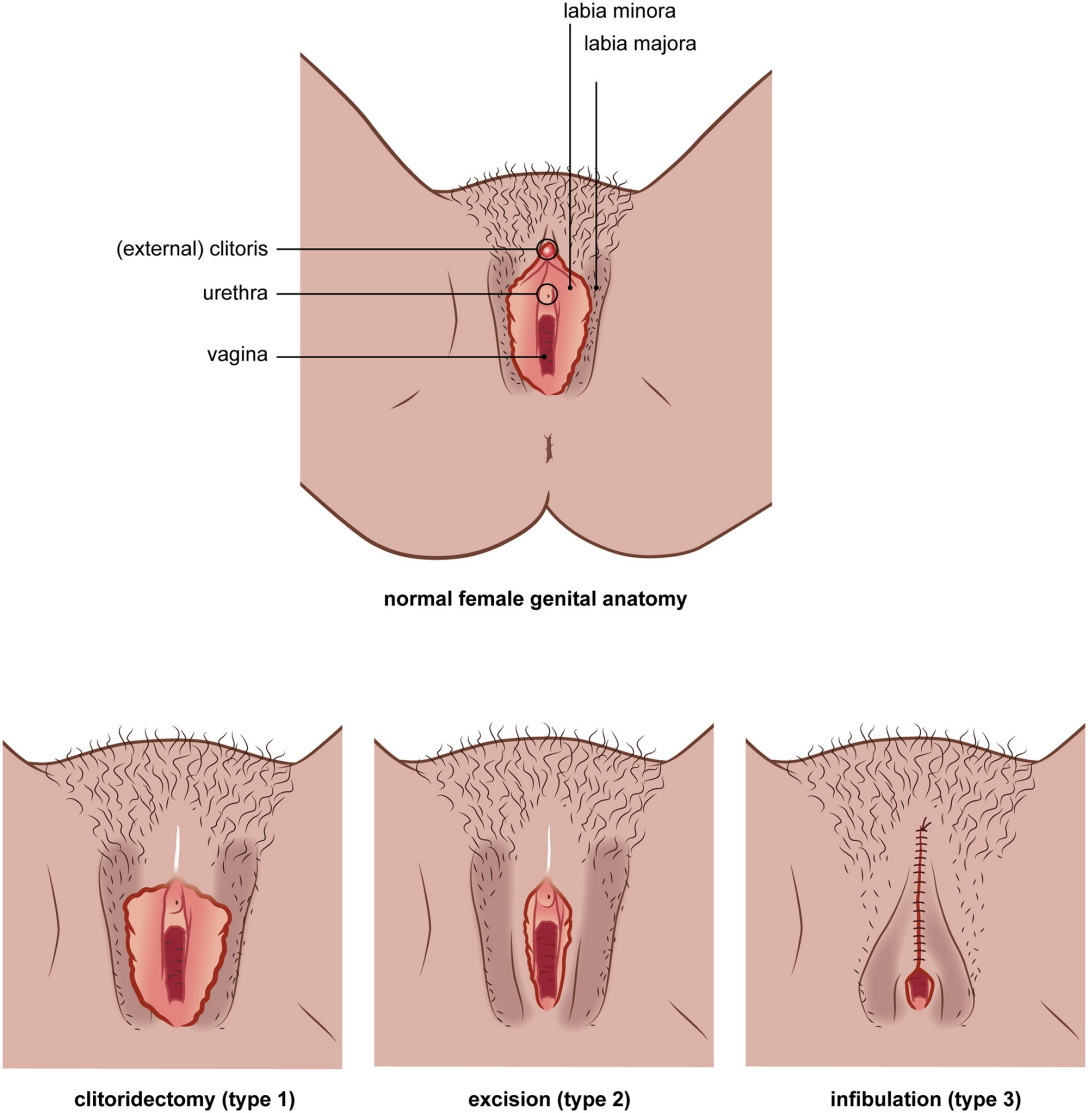
World Health Organization classification of female genital mutilation or cutting. Type I refers to excision of the prepuce, with or without excision of part or all of the clitoris. Type II refers to excision of the clitoris with partial or total removal of the labia minora. Type III refers to excision of part or all of the external genitalia and stitching or narrowing of the vaginal opening (infibulation). Type IV includes all other harmful procedures to the female genitalia for nonmedical purposes, e.g., pricking, piercing, incising, scraping and cauterizing the genital area [4, 5].

In the Netherlands, as in many other Western countries, the prevalence of women who have undergone female genital mutilation or cutting (FGM/C) is on the rise due to increased migration from the countries where FGM/C is traditionally practiced [1, 2]. FGM/C is defined as harm to the female genitalia, including those practices whereby the external female genitalia are either partially or completely removed for nonmedical reasons [3]. The WHO has classified four main types of FGM/C [4] (see Fig 1).

According to estimations, approximately 200 million women and girls worldwide have undergone FGM/C [6]. It is performed in countries across Africa, Asia and the Middle East and is a tradition with various meanings and local characteristics, often seen as a cultural, religious and/or social norm [7]. The Netherlands has had a zero-tolerance policy regarding FGM/C since the introduction of a law in 1993, meaning that practicing any form of FGM/C is forbidden on a girl who is resident of the Netherlands—performed in the Netherlands or abroad [8–11]. Recent research estimated that in 2018, approximately 41,000 women living in the Netherlands had undergone FGM/C, and approximately 37% of them had Type III FGM/C. The majority of them are migrants or their daughters from Somalia, Egypt, Ethiopia, Eritrea, Sudan and northern Iraq [12].

FGM/C can cause physical, mental and psychosocial health problems, including both short-term complications of the procedure and long-term complications. The latter problems range from urinary to sexual issues and problems related to childbirth [5, 13–18].

Finding adequate care, however, has proven to be difficult for these women due to lack of awareness, expertise and empathy among healthcare practitioners but also because women do not attribute their health problems to FGM/C [19–21]. Furthermore, misinterpretation of the Dutch law led some women to believe that being circumcised was punishable, which prevented them from seeking healthcare [1]. Most previous studies have focused on the gynecologic and obstetric healthcare experiences of FGM/C women [18, 19, 22, 23], and very little is known about women’s experiences with primary healthcare.

In the Netherlands, the general practitioner (GP) is the first point of entry to and gatekeeper of the healthcare system. All people who have obligatory health insurance are enlisted with a GP; specialist care is only accessible through referral by the GP. Therefore, the GP is often the first to encounter the health problems of women with FGM/C. They could play a vital role in informing women with FGM/C. However, no study exists to explore the experiences of women with FGM/C in general practices in the Netherlands. The aim of this study is to look into the experiences of women with FGM/C in Dutch general practice.

## Methods

### Study design

A qualitative study design was used to obtain an in-depth understanding of the healthcare experiences from the perspective of women who have undergone FGM/C with general practitioners (GPs) in the Netherlands. The study was conducted between May and August 2017. The study was approved by the Maastricht University FHML Research Ethics Committee (Application nr. 2017/04).

### Recruitment and study population

The study population consisted of women older than 18 years living in the Netherlands who had undergone FGM/C and had sufficient fluency in Dutch or English. Sampling was purposeful, aiming for maximum variation regarding age, country of origin, duration of residence in the Netherlands and education level. Recruitment was done using a trusted intermediary, who approached eligible women by phone, after which they then received an informational letter explaining the purpose of the study. If they agreed to be interviewed, the study was again explained orally, after which participants signed the informed consent form. Women’s FGM/C type was not known prior to interviewing; the FGM/C types were classified by the women themselves using drawings provided in the interview as an aid (see Fig 1). Participants were recruited and interviewed until no new information was mentioned in the interviews and data saturation was reached.

### Data collection

Data were collected through individual, face-to-face, semistructured interviews. The interview followed a topic list based on the following frameworks: the Illness Perception Model (IPM), which explains the process of a person’s illness perception including health- and illness- awareness and definition [24], and Arthur Kleinman’s Core Clinical Functions and Explanatory Model, which addresses the influence of culture on an individual’s understanding of illness and disease [25]. The topic list contained questions on FGM/C, health complaints, the possible link between health complaints and FGM/C and women’s experiences with their GP. The interview guide was tested by a Somali intermediary fluent in both Dutch and English to ensure appropriateness of language and cultural sensitivity.

Each interview took place with EA or EN. The two (female) interviewers were Master’s students in global health who practiced their internship at Pharos, national knowledge center on Female Genital Mutilation in the Netherlands. The interview took place in Dutch or English depending on participants preference and in a setting of the participants’ choice, most often the home setting, which helped ensure a safe environment in which to speak freely. Each interview had an average length of 50 minutes and was recorded using a mobile recording device. Participants received a gift voucher, along with contact details of a physician in case women had any questions or problems after the interview.

### Data analysis

All data were anonymized, and recordings were transcribed verbatim by one of the researchers. To ensure reliability, interviews were coded and analyzed thematically by both researchers independently using Nvivo (version 10) as well as manually. An open coding system was applied for thematic analysis, developed in line with the theoretical frameworks. The coding system, preliminary results and any discrepancies during coding and analysis were discussed with the research team to ensure credibility. Based on this, codes were adapted or added. Quotations from Dutch interviews were eventually translated into English.

## Results

In total, 16 women were interviewed, varying in age, country of origin, duration of residence in the Netherlands and educational level (see Table 1). All women had consulted a GP in the Netherlands at least once, not necessarily related to FGM/C. Ten of the women had discussed FGM/C with their GP; six of the women had not.

**Table 1:** Characteristics of study population.

Characteristics	*n* = 16
Age (years)	35 (median)
	23–56 (range)
Country of origin	
Egypt	2
Ethiopia	1
Guinea	2
Nigeria	1
Sierra Leone	2
Sudan	4
Somalia	4
Education	
Primary	2
Secondary	6
Tertiary	8
Duration of residence in the Netherlands (years)	
< 5	4
6–10	2
> 10	10
Number of children	
None	2
1	4
2	4
3	5
4	1
FGM/C types	
Type I	6
Type II	3
Type III	7
Type IV	0

### FGM/C as a health issue

Women’s views of FGM/C had changed over time, when compared to what FGM/C meant to them in their country of origin. Before migrating to the Netherlands, they did not seem to consider themselves different, while now they felt that they were being viewed as deviant by the Dutch society.

*“[…] Before*, *you do not feel shy because you are circumcised*, *but now you feel shy*, *a little bit*, *because you know the difference*. *I now know*, *I am not a complete woman*. *It is like that*. *But before*, *we did not know*, *we thought we are all the same*, *we did not know the difference*.*”*

(R9)

Women also seemed to start wondering whether other health issues they were having, which they before considered normal, were also abnormal. They mentioned health issues such as problems with menstruation, urination, sex, pregnancy and childbirth.

*“I never knew that circumcision can cause so many problems*, *issues that you do not know about*. *Because in Africa or as an African woman*, *we feel that it is normal to be circumcised*. *Until you come across those people [who have not been circumcised]*, *you do not know the difference*.*”*

(R9)

and

*“Yes*, *circumcision makes childbirth harder than for a normal woman*. *Also*, *with the menstruation*, *everything [is harder]*. *Everything*!*”*

(R4)

### Expectations of healthcare related to FGM/C

Some participants did not expect or want FGM/C-related care from their GP at all, either because they did not need any care at all or because they believed that their GP was not the one who could help them.

*“It is a very unpleasant feeling*, *yes*. *And you cannot change that*, *that is the problem here*. *You cannot change it; you just have to deal with it*. *[…] I am thinking about the operation with the doctor to make a pretty shape or something [reconstructive surgery]*, *but I do not know if it will help or not*. *[…] The things they removed; they will never come back*.*”*

(R14)

and

*[Question*: *Have you thought about going to see a doctor with this problem*?*]*

*“No*, *I have the feeling that they cannot do anything*. *They can reconstruct it*, *but I have the feeling that it will never be the same again*.*”*

(R2)

and

*“[…] I think if I talk with my general practitioner about a problem with the circumcision*, *it will not help me*. *I think talking with a specialist will be more effective for me*.*”*

(R6)

Other women, who indicated they would like FGM/C-related care from the GP, appeared to be unsure about how to ask for this care. Some of them felt uncomfortable or ashamed to discuss FGM/C or related sensitive health issues. Moreover, women indicated that they found it difficult to bring up FGM/C themselves with their GP.

*“I think that the doctor will think that I have a low level of thinking [intelligence]*. *I know that women are ashamed*, *maybe that is why*. *For me too*, *I am always ashamed*.*”*

(R4)

and

*“But if you expect a Somali woman*, *or to be honest*, *any Islamic woman*, *to come and tell you about her issues*, *you will not get her to tell you*. *So then she just keeps on coming and then the wrong diagnoses are made*, *but you will not get to the point*. *Nothing will happen*.*”*

(R5)

Most women expressed a strong preference for the GP to be the one to initiate a conversation on the topic. Many respondents were convinced that care would be improved if the GP would ask them about FGM/C.

*“Those are the things that I then did not dare to discuss with the general practitioner*. *But I do think that*, *well*, *if I would have been giving a key word or the question ‘are you circumcised*?*’ that I would have received better help*.*’”*

(R5)

Furthermore, many women considered GPs to have a lack of knowledge about FGM/C and therefore to be unable to help them with their health problems. Many women said they had to explain things about FGM/C to their GPs. Some women felt they could not be helped by someone who did not know and understand them and their culture.

*“How did the general practitioner react to the circumcision*? *He was shocked*. *And he did not know*, *he didn’t know anything about it*. *He may have heard of it*, *but he did not know exactly what circumcision was*. *He did not know what kind of issues women who have been circumcised have*. *He also did not know that some women have issues and other women do not*. *So*, *I told him and explained to him that it depends on the type of circumcision*, *but he does not know*.*”*

(R1)

### Experiences with healthcare related to FGM/C

In some cases, such as those described above, the women thought the GP was shocked when (s)he found out a patient had undergone FGM/C. However, they did not seem to experience this as negative and understood the GP’s reaction.

*“She [GP] asked me*, *“Are you by any chance circumcised*?*” I said*, *“Yes*.*” For me it was the most normal thing in the world*. *And then she said*, *“Can I examine you*?*” So*, *then I said*, *“Yes*, *why not*.*” So*, *she examined me and she was shocked*.*”*

(R7)

None of the women mentioned their GP’s having a negative attitude or opinion towards FGM/C.

### Satisfaction with healthcare related to FGM/C

Women felt pressured by the limited consultation time or felt that the GP was rushed and therefore did not give them the attention they needed.

*“What did you think of the visit to the general practitioner*? *How was the appointment*?*”*

(Interviewer)

*“It was not good*! *Because it was less than three minutes*. *I explained to her [GP] what was going on and she said that everything was all right*. *But she did not even do a check-up*, *because she does not have the time*, *whilst she could not have known that in such a short time*.*”*

(R2)

A point of dissatisfaction was the actual care provided by some GPs. Some women felt the care was not relevant to their health issues. Another point of dissatisfaction was the Dutch healthcare system.

*“[…] and then I went to the general practitioner*. *I did not get any medicine*. *I do not know how it works in the Netherlands*, *but they always recommend you to take paracetamol [acetaminophen]*. *But you just go to the general practitioner to check there is nothing wrong*, *but you never get anything for the pain*.*”*

(R3)

and

*“[…] In the Netherlands everything is just a process*, *everything is really complicated*. *When you get in the medical mill*, *you get into the process*. *You go to the general practitioner; they send you to there and there*. *Or if you have to make an appointment*, *you have to wait for months or travel far and you just do not feel like doing that*.*”*

(R3)

There were also examples of women who were satisfied with the care they received from their GP. Important aspects of satisfaction were when the GP gave them the impression (s)he understood them and when the GP took enough time to understand their health issues.

*“[…] And I really felt that she was concerned about me and sympathized with me*. *I think she must have imagined*: *what if I would have been her*, *what then*? *[…] I was lucky*, *it is really true*. *It all had to do with the general practitioner*. *I do not think they are all like that*.*”*

(R7)

The women most satisfied with their GP were those to whom the GP had provided care for nonmedical issues related to FGM/C. For example, one woman received, upon her request, a letter from her GP to show her future husband.

*“[…] So*, *I had the reconstruction surgery*, *and luckily the general practitioner gave me papers in case I got married after*, *to explain why I had the surgery*. *Because it is not part of our culture*. *And luckily*, *everything turned out all right*. *[…]”*

(R5)

Being asked if they preferred a male or a female GP, some respondents indicated a preference for a female GP, as they felt it was easier to discuss women’s issues with another woman.

*“Depends on the issues that I am having*. *If they are real urgent issues*, *then I will go [to any doctor]*. *But you feel more at ease telling your issues to a woman*. *[…] So*, *from my side*, *I find it better to speak to discuss it with a woman*. *Because if you would have come today and you would have been a man*, *then I would have not been as open*. *That is personal*, *you know*.*”*

(R3)

Other women did not seem to mind whether their GP was male or female, as long as the care they received was satisfactory.

*“No*, *a doctor is a doctor; for me it does not make a difference*.*”*

(R1)

## Discussion

The women in our study experienced a range of medical problems that they associated with the Female Genital Mutilation/Cutting (FGM/C) they had undergone, but not all of them sought medical care for these problems with their general practitioner (GP). Some of them did not think care would resolve their problems, and others did not think Dutch GPs would have sufficient knowledge of FGM/C or did not know how to discuss FGM/C. All women preferred the GP to proactively ask about FGM/C. In general, women thought positively about their GP, but overall, they were dissatisfied with Dutch healthcare and GP care, in particular with the limited time available during consultations. GPs who offered care beyond biomedical problems were valued most.

As discussed by Evans et al. [26], due to the sensitive and taboo nature of FGM/C, it is difficult for both women and healthcare providers to talk about FGM/C. Thus, it is not surprising that women find it difficult to bring this topic up themselves in discussion with their GP or to reveal their intimate parts, which can also lead to reluctance in seeking healthcare, for instance during pregnancy [16]. Especially when they have experienced a shocked reaction from a doctor examining their mutilated vulva, or a disapproving attitude from their new society, women often develop shame about FGM after their migration [16, 27]. Most of our participants have learned upon arrival and during their resettlement in the Netherlands that not all women have undergone FGM/C and that the practice is not the norm globally. Hence, they have started to feel ‘abnormal’ and attributed their medical problems to FGM/C. This corresponds with other studies, in which women reported discovering through migration that not all women have FGM/C and questioned the practice [16, 28, 29]. A study in the Netherlands examining the psychosocial consequences of FGM/C concluded many women to be chronically suffering from mental and psychosocial problems and showed that women had become more aware of the negative consequences of FGM/C after migrating to the Netherlands, showing a shift in their perceptions [16].

Lack of expertise among doctors, alongside inadequate assessment of the patients’ problems, was found to be a main barrier to good quality of healthcare in other studies [21, 30, 31]. In specialized FGM/C care settings, such as the FGM/C clinic in London [32] or the specialized consultation facilities in the Netherlands [21], women seem to have better experiences with the care provided, thus emphasizing the importance of appropriate healthcare, information and support [21, 27, 32].

The need for proper assessment, and thus the active asking about FGM/C by the GP, was also expressed by the women in our study. Such a proactive approach to sensitive topics is well-known to be effective in GP care [33], as long as it is done in a respectful, culturally sensitive way [34]. The finding that GP care was most valued if it extended beyond the biomedical domain is in line with the strong preference of patients for, and positive effects of, person-centered care [35].

## Strengths and limitations

To the best of our knowledge, this is the first study on FGM/C experiences in general practice in the Netherlands. There are some limitations to take into account. While the use of a trusted migrant organization as intermediary was necessary for recruitment, this could have led to bias, as this organization is known to be opposed to FGM/C. Interviewing a different group of women could provide alternative views and opinions on FGM/C and possibly also on healthcare experiences with GPs. In addition, only women with sufficient fluency in Dutch or English were recruited, which excluded migrant women who, due to language barriers, might encounter even more difficulties in accessing good quality of care.

## Recommendations for future research and for practice

In general, GPs may benefit from training on diversity, cross-cultural competence and communication skills related to FGM/C prevention and management. A FGM/C Conversation Guide and the guidelines on the management of FGM/C [36], which is developed by the Dutch Society of Obstetrics and Gynecology (NVOG) [36] could also provide a useful support to healthcare providers. Insights into GPs’ knowledge of and experiences with FGM/C related care could help to develop effective educational curricula for medical students and GPs (i.e., trainees) on FGM/C.

Following the suggestions of the women in our study, GPs (and other healthcare professionals) should address the topic of FGM proactively. For instance, the GP could ask about FGM/C during their first consultation with women from FGM/C-practicing countries. This could help to ‘normalize’ the topic and convince women they can freely discuss this with their GP.

To develop the trusted relationship needed to discuss sensitive topics and provide culturally sensitive person-centered-care, sufficient time during consultations, generally more than the usual 10-minute slot, is needed.

## Conclusion

The women with FGM/C experienced a range of health problems; however, not all of them sought medical care for these problems. On one hand, they had difficulty discussing such a sensitive topic with the GP, and on the other hand, they perceived GPs to have insufficient knowledge of FGM/C to provide them with adequate care. To improve the healthcare experience of women with FGM/C in general practice in the Netherlands, it is advisable that GPs take a proactive attitude in discussing such sensitive topics with their patients. In addition, to develop the trusted relationship needed to discuss sensitive topics and provide culturally sensitive person-centered care, sufficient time during consultations is needed.
